# Comparative Clinical Study of Different Multiplex Real Time PCR Strategies for the Simultaneous Differential Diagnosis between Extrapulmonary Tuberculosis and Focal Complications of Brucellosis

**DOI:** 10.1371/journal.pntd.0002593

**Published:** 2013-12-12

**Authors:** Rocio Sanjuan-Jimenez, Pilar Morata, Pilar Bermúdez, M. José Bravo, Juan D. Colmenero

**Affiliations:** 1 Biochemistry, Molecular Biology and Immunology Department, Faculty of Medicine, University of Malaga, Malaga, Spain; 2 Microbiology Service, Carlos Haya University Hospital, Malaga, Spain; 3 Infectious Diseases Service, Carlos Haya University Hospital, Malaga, Spain; University of California San Diego School of Medicine, United States of America

## Abstract

**Background:**

Both brucellosis and tuberculosis are chronic-debilitating systemic granulomatous diseases with a high incidence in many countries in Africa, Central and South America, the Middle East and the Indian subcontinent. Certain focal complications of brucellosis and extrapulmonary tuberculosis are very difficult to differentiate clinically, biologically and radiologically. As the conventional microbiological methods for the diagnosis of the two diseases have many limitations, as well as being time-consuming, multiplex real time PCR (M RT-PCR) could be a promising and practical approach to hasten the differential diagnosis and improve prognosis.

**Methodology/Principal Findings:**

We designed a SYBR Green single-tube multiplex real-time PCR protocol targeting *bcsp31* and the *IS711* sequence detecting all pathogenic species and biovars of *Brucella* genus, the *IS6110* sequence detecting *Mycobacterium* genus, and the intergenic region senX3-regX3 specifically detecting *Mycobacterium tuberculosis complex*. The diagnostic yield of the M RT-PCR with the three pairs of resultant amplicons was then analyzed in 91 clinical samples corresponding to 30 patients with focal complications of brucellosis, 24 patients with extrapulmonary tuberculosis, and 36 patients (Control Group) with different infectious, autoimmune or neoplastic diseases. Thirty-five patients had vertebral osteomyelitis, 21 subacute or chronic meningitis or meningoencephalitis, 13 liver or splenic abscess, eight orchiepididymitis, seven subacute or chronic arthritis, and the remaining seven samples were from different locations. Of the three pairs of amplicons (senX3-regX3+ bcsp3, senX3-regX3+ IS711 and IS6110+ IS711) only senX3-regX3+ IS711 was 100% specific for both the *Brucella* genus and *M. tuberculosis complex*. For all the clinical samples studied, the overall sensitivity, specificity, and positive and negative predictive values of the M RT-PCR assay were 89.1%, 100%, 85.7% and 100%, respectively, with an accuracy of 93.4%, (95% CI, 88.3—96.5%).

**Conclusions/Significance:**

In this study, a M RT-PCR strategy with species-specific primers based on senX3-regX3+IS711 sequences proved to be a sensitive and specific test, useful for the highly efficient detection of *M. tuberculosis* and *Brucella* spp in very different clinical samples. It thus represents an advance in the differential diagnosis between some forms of extrapulmonary tuberculosis and focal complications of brucellosis.

## Author Summary

Both brucellosis and tuberculosis are systemic infections which may involve any organ. When they affect specific locations, extrapulmonary tuberculosis and brucellosis cause symptoms that are very difficult to differentiate clinically. Mycobacterium tuberculosis complex and Brucella spp are slow-growing microorganisms whose culture and isolation require several days to weeks. Methods based on the polymerase chain reaction (PCR) have proven more sensitive than conventional culture, for both extrapulmonary tuberculosis and focal complications of brucellosis. Multiplex real time PCR is a variant of PCR in which two or more target sequences can be simultaneously amplified in a single tube. We developed and evaluated the results of several multiplex real-time PCR strategies for the rapid differential diagnosis between extrapulmonary tuberculosis and focal complications of brucellosis. Multiplex real-time PCR targeting of SenX3-RegX3+IS711 sequences showed a sensitivity of 89.1% and a specificity of 100% when applied to 91 clinical specimens. These findings provide solid evidence suggesting that multiplex real-time PCR could be a useful tool to reduce the time required for the differential diagnosis between extrapulmonary tuberculosis and complicated brucellosis, thereby improving prognosis.

## Introduction

Brucellosis remains one of the most widespread anthropozoonoses in the world, especially in the Mediterranean basin, the Middle East, India, Mexico and some countries of Central and South America [1]. Much evidence supports the conclusion that in countries without strong health systems, official data likely underestimate the true burden [2]. The high morbidity associated with brucellosis, together with its prolonged course and great tendency to produce relapses account for an important consumption of health care resources [3], [4].

The global burden of tuberculosis (TBC) remains enormous [5]. Recent data in the WHO Global Tuberculosis Report 2012 confirm that TBC remains a major infectious killer today. In 2011, there were an estimated 8.7 million new cases and 1.4 million people died from TBC [6].

Like TBC, brucellosis can cause focal complications in any organ or system. The larger studies place the rate of focal complications of brucellosis at around 25–35% of all cases [3], [7], [8], similar to the rate of extrapulmonary complications in TBC, 15–40% [9]. Moreover, whilst in many countries there has been a reduction in the overall incidence of pulmonary tuberculosis, the number of extrapulmonary tuberculosis cases has increased in some industrialized countries [10]–[13].

When tuberculosis or brucellosis affect specific sites, e.g., the CNS, or osteoarticular or genitourinary systems, the differential diagnosis between the two entities is virtually impossible based solely on clinical, haematological, biochemical or imaging studies. Furthermore, as both tuberculosis and brucellosis are granulomatous diseases, the pathological findings of focal complications of brucellosis and extrapulmonary tuberculosis can be very similar.

Both *Mycobacterium tuberculosis* complex (MTC) and *Brucella* spp are slow-growing microorganisms. Classical methods for determining the presence of these microorganisms are time-consuming and labor-intensive. Hence, molecular methods, which offer speed, sensitivity and specificity, have been developed to address this problem. Multiplex real time PCR (M RT-PCR) is increasingly used in various fields of microbiology for the rapid differentiation of microbial species involved in specific syndromes [14]–[16].

Our group has shown that M RT-PCR is a useful strategy for the rapid differential diagnosis between extrapulmonary tuberculosis and brucellosis when they affect specific locations [17]. Later, we simplified the technique to make it more accessible to any clinical laboratory [18]. This study compared experimentally, in both monoplex and multiplex forms, the PCR combinations of three different targets for each microorganism, optimizing and simplifying the technique using SYBR Green, determining the sensitivity and reproducibility in a small sample of patients.

The aim of the present study was to analyze comparatively the diagnostic yield of different strategies of M RT-PCR in a very representative sample of patients with focal complications of brucellosis or extrapulmonary tuberculosis and assessed the analytical specificity against a wide panel of microorganisms that included most of the non-tuberculous Mycobacteria related with human diseases, and the most important species and biovars of Brucella.

## Methods

### Study population and clinical samples

The study included 91 non-respiratory samples from 90 patients aged >14 years. Of the 90 patients, 30 had focal complications of brucellosis, 24 had extrapulmonary tuberculosis and 36 (Control Group) had various different infectious, autoimmune or neoplastic diseases in which the treating physician initially raised the possibility of extrapulmonary tuberculosis or brucellosis in the differential diagnosis. One patient with brucellosis provided two different samples from two simultaneous focal complications.

### Ethics statement

The aims of the study were communicated to the participants and a written informed consent form was signed before the inclusion to the study. Whenever the subjects were minors the informed consent was given by the parents or legal guardians as appropriate in each case. The use of samples for research was approved by the Ethics Committees of Malaga University and Carlos Haya University Hospital, Malaga, Spain.

### Tuberculosis and brucellosis diagnostic criteria

The diagnosis of tuberculosis was established according to one of the following criteria: first, isolation of *M. tuberculosis* or second, the presence of caseating granulomas, with or without acid-fast bacilli in a patient with a compatible clinical picture and good therapeutic response to antituberculous treatment. The diagnosis of brucellosis was based on isolation of *Brucella* spp. in blood or any other body fluid or tissue sample or, second, the presence of a compatible clinical picture together with the demonstration of specific antibodies at significant titers or seroconversion. Significant titers were considered to be a standard agglutination test (SAT) ≥1/160 or immunocapture agglutination test ≥1/320.

### Bacterial strains, culture media and growth conditions

The specificity of the M RT-PCR assays was assessed from a widely representative panel of the various biovarieties of *Brucella* spp and phylogenetically or serologically related microorganisms, species belonging to MTC and strains of non-tuberculous mycobacteria (NTM) from the collection and clinical isolates of the Microbiology Laboratory at Carlos Haya University Hospital (HCH). The MTC strains were selected from the American Type Culture Collection (ATCC) and clinical samples from the Microbiology Laboratory at HCH. All the isolates of the clinical samples were later characterized at the Mycobacterium reference laboratory, at the Instituto de Salud Carlos III, Madrid, Spain. The different strains of NTM and *Nocardia* spp were supplied by the Spanish Type Culture Collection (CECT) or were clinical isolates from HCH. The strains of mycobacteria were cultured in Lowenstein-Jensen medium and incubated at 37°C for 2–4 weeks, in order to obtain sufficient bacterial growth for the later extraction of genomic DNA. The strains of *Brucella* spp were provided by the Department of Microbiology, of the Faculty of Medicine, University of Valladolid (Spain), except for the vaccine strains B-19 and Rev 1, which were supplied by the Andalusian Government Ministry of Agriculture and Fisheries. These strains were cultured in Brucella agar and incubated at 37°C in an atmosphere containing 5% CO_2_ for 48 hours. Genomic DNA from bacterial strains serologically or phylogenetically related with *Brucella* were provided by the CECT, except for the species *Ochrobactrum intermedium*, kindly provided by the Faculty of Medicine of the University of Navarra, Spain. All procedures were performed in a biosafety cabinet class II B3.

### DNA extraction

All samples destined for M RT-PCR were maintained at −20°C until processing. The amount or volume used varied depending on the type of sample. To monitor contamination, negative controls were included during each DNA extraction procedure. DNA was extracted using the Quiamp DNA Mini (Qiagen, UK). Prior to DNA extraction, homogenized samples from the different tissues, CSF, synovial fluid, urine, purulent collections and strains were resuspended in 1 ml of molecular biology water, mixed and centrifuged at 15.000× g for 10 min. The supernatant was discarded and the pellet was resuspended with the volume of buffer outlined in the manufacturer's instructions. DNA pellets were resuspended in 50 µl molecular biology water and stored at 4°C until use. The concentration and purity of DNA were estimated by measuring the absorbance at 260 and 280 nm with a ND-1000 spectrophotometer (Nanodrop ThermoFisher, USA).

### Primer design and Multiplex Real Time PCR assay conditions

For detection of members of MTC, the primer sets IS6110f/IS6110r (5′ TCAAGGAGCACATCAGCC3′/5′TCACGGTTCAGGGTTAGC3′) and M1f/M3r (5′CGGCTAATCACGACGGCAC3′/5′CTCTTCCTCTCGTTGTGACCTGTT 3′) were used to amplify 82 and 164 bp fragments of IS6110 and senX3-regX3, respectively. For *Brucella*, fragments of 152 and 142 bp of the bcsp31 gene and IS711 were amplified using primers bcsp31f/bcsp31r (5′ GCATTCTTCACATCCAGG 3′/5′ CACCGCATTCCATTATTCT 3′) and IS711f/IS711r (5′ TACAAGGAACGCCATCAGA 3′/5′ GCATTCAACGCAACCAGA) [18]. The three real time reactions were monitored using a Light-Cycler 2.0 (Roche Diagnostic, Indianapolis, IN) with the LC FastStart DNA Master SYBR-Green I kit (Roche Molecular Biochemicals, Mannheim, Germany). The M RT-PCRs for MTC and *Brucella* were performed as described previously [18]. Briefly, the mixture included 1× master mix, 3–3.5 mM MgCl_2_, 0.5 µM primers, variable concentrations of DNA as template (150–250 ng depending on type of sample analyzed) and nuclease free dH_2_O adjusted to a final volume of 20 µl. Each run included positive controls consisting of dilutions of *Brucella* spp and MTC DNA, and negative controls with all the elements of the reaction mixture except template DNA. The reactions were cycled 45 times, after an initial hold at 95°C for 10 min, between 95°C for 10 s, 60°C for 5 s, and 72°C for 6 s with programmed transitions of 20°C/s. The melting curves were acquired on the SYBR channel by heating momentarily at 95°C, cooling to 65°C and collecting fluorescence continuously at a ramping rate of 0.1°C/s until 95°C. To minimize experimental variability the Ct values, the threshold cycle where the fluorescence signal rises significantly above background in the exponential phase of the amplification, were determined by the second derivative maximum method. In order to avoid potential observer bias, the clinical and microbiological diagnoses of the patients were unknown to the technician who performed the M RT-PCR assay.

### Primer specificity

The specificity of the primers was first tested in silico using the BLASTn program in order to prevent non-specific amplifications. The analytical specificity was then tested against the 59 microorganisms listed in Table 1.

**Table 1:** M RT-PCR results with DNA from different microorganism included in this study.

Species	Strain	Origin	M RT-PCR								
			senX3-regX3+bcsp31			senX3-regX3+IS711			IS6110+IS711		
			MTC	*Brucella*	Tm (°C)	MTC	*Brucella*	Tm (°C)	MTC	*Brucella*	Tm (°C)
***Mycobacterium strains***											
**MTC**											
*M. tuberculosis*	H37Rv	ATCC	+	−	89.32±0.02	+	−	89.34±0.02	+	−	87.31±0.04
*M. caprae*	1040	HCH	+	−	89.11±0.29	+	−	89.06±0.04	+	−	87.32±0.38
*M. caprae*		HCH	+	−	89.21±0.02	+	−	89.75±0.08	+	−	87.47±0.19
*M. africanum*	25420	ATCC	+	−	89.52±0.44	+	−	89.76±0.03	+	−	87.83±0.02
*M. africanum*		HCH	+	−	89.38±0.36	+	−	89.07±0.01	+	−	87.72±0.03
*M. bovis* BCG	Pasteur	ATCC	+	−	90.18±0.37	+	−	89.48±0.14	+	−	87.75±0.22
*M. bovis*	19210	ATCC	+	−	89.32±0.14	+	−	89.12±0.03	+	−	87.36±0.34
*M. bovis*	XDR	HCH	+	−	90.12±0.06	+	−	89.17±0.09	+	−	87.81±0.03
*M. microti*	8710	ATCC	+	−	89.06±0.02	+	−	89.42±0.09	+	−	87.52±0.05
*M. pinnipedii*	13288	ATCC	+	−	89.27±0.08	+	−	89.40±0.37	+	−	87.82±0.18
**Genetically related bacteria**											
**NTM**											
*M. avium*	1062	ATCC	−	−		−	−		−	−	
*M. xenopi*		HCH	−	−		−	−		−	−	
*M. kansasii*		HCH	−	−		−	−		−	−	
*M. chelonae*		HCH	−	−		−	−		−	−	
*M. gordonae*		HCH	−	−		−	−		−	−	
*M. fortuitum*		HCH	−	−		−	−		+	−	86.57±0.17
*M. scrofulaceum*		HCH	−	−		−	−		+	−	87.00±0.01
*M. szulgai*		HCH	−	−		−	−		−	−	
*M. marinum*	7091	CECT	−	−		−	−		−	−	
*M. celatum*	342	ATCC	−	−		−	−		−	−	
*M. intracellulare*		HCH	−	−		−	−		+	−	87.16±0.16
*M. simiae*		HCH	−	−		−	−		+	−	87.01±0.08
*M. smegmatis*	3017	CECT	−	−		−	−		−	−	
*M. flavencens*	3027	CECT	−	−		−	−		−	−	
*M.phlei*	3016	CECT	−	−		−	−		−	−	
*M.brumae*	3022		−	−		−	−		−	−	
*M. abscessus*		HCH	−	−		−	−		−	−	
*M. mucogenicum*		HCH	−	−		−	−		−	−	
*M. peregrinum*		HCH	−	−		−	−		−	−	
**Other acid-fast microrganism**											
*Nocardia* spp		HCH	−	−		−	−		−	−	
***Brucella*** ** spp (22)**											
*B. melitensis* biovar 1	16 M	FMV	−	+	86.84±0.10	−	+	84.45±0.10	−	+	84.25±0.60
*B. melitensis* biovar 1	Rev 1	CAJA	−	+	86.24±0.06	−	+	84.78±0.26	−	+	84.42±0.07
*B. melitensis* biovar 2	63/9	FMV	−	+	86.08±0.10	−	+	84.72±0.09	−	+	84.77±0.07
*B. melitensis* biovar 2	AC	FMV	−	+	86.62±0.40	−	+	84.72±0.20	−	+	84.40±0.21
*B. melitensis* biovar 3	Ether	FMV	−	+	86.95±0.04	−	+	84.49±0.07	−	+	84.54±0.09
*B. abortus* biovar 1	AC	FMV	−	+	86.26±0.02	−	+	84.12±0.11	−	+	84.77±0.39
*B. abortus* biovar 1	B19	CAJA	−	+	86.49±0.08	−	+	84.67±0.21	−	+	84.85±0.73
*B. abortus* biovar 2	86/8/59	FMV	−	+	86.56±0.37	−	+	84.67±0.21	−	+	84.48±0.02
*B. abortus* biovar 3	Tulya	FMV	−	+	86.31±0.22	−	+	84.46±0.14	−	+	84.37±0.04
*B. abortus* biovar 4	292	FMV	−	+	86.22±0.03	−	+	84.30±0.04	−	+	84.40±0.01
*B. abortus* biovar 5	B3196	FMV	−	+	86.32±0.11	−	+	84.37±0.24	−	+	84.48±0.21
*B. abortus* biovar 6	870	FMV	−	+	87.22±0.35	−	+	84.29±0.50	−	+	84.76±0.47
*B. abortus* biovar 7	63/75	FMV	−	+	86.79±0.64	−	+	84.37±0.31	−	+	84.55±0.25
*B. abortus* biovar 9	C/68	FMV	−	+	86.81±0.53	−	+	84.47±0.22	−	+	84.92±0.35
*B. suis* biovar 1	10036	FMV	−	+	86.76±0.18	−	+	84.89±0.19	−	+	84.28±0.56
*B. suis* biovar 2	10510	FMV	−	+	86.89±0.03	−	+	84.74±0.43	−	+	84.83±0.20
*B. suis* biovar 3	10511	FMV	−	+	86.73±0.02	−	+	84.60±0.02	−	+	84.72±0.09
*B. suis* biovar 4	40	FMV	−	+	86.35±0.27	−	+	84.42±0.72	−	+	84.67±0.36
*B. suis* biovar 5	10980	FMV	−	+	87.18±0.01	−	+	84.68±0.67	−	+	84.86±0.04
*B. neotomae*	10084	FMV	−	+	86.96±0.44	−	+	84.59±0.01	−	+	85.05±0.32
*B. ovis*	Reo198	FMV	−	+	86.96±0.07	−	+	84.96±0.10	−	+	84.68±0.05
*B. canis*	10854	FMV	−	+	86.52±0.02	−	+	84.36±0.16	−	+	84.69±0.16
**Antigenically related bacteria**											
*Escherichia coli*	O157:H7	CECT	−	−		−	−		−	−	
*Moraxella osloensis*	460	CECT	−	−		−	−		−	−	
*Pasteurella multocida*	962	CECT	−	−		−	−		−	−	
*Yersinia enterocolitica*	O:9	CECT	−	−		−	−		−	−	
*Vibrio cholerae*	Inaba	CECT	−	−		−	−		−	−	
**Genetically related bacteria**											
*Ochrobactrum anthropi*	4426T	CECT	−	+	86.84±0.11	−	−		−	−	
*Ochrobactrum intermedium*	3301	FMN	−	+	86.69±0.71	−	−		−	−	

### Sequencing of M RT-PCR product

To confirm the identities of the amplified fragments, some of the strains used as positive controls of *Brucella* spp and MTC and different clinical samples were sequenced. The ABI PRISM Big Dye Terminator Cycle sequencing reaction kit v. 3.0 (Applied Biosystems, Madrid, Spain) was used for the sequencing analysis, by capillary electrophoresis, in an ABI PRISM, model 3100 automated sequencer (Applied Biosystems).

### Statistical analysis

Quantitative variables are represented as mean ± standard deviation and qualitative variables as percentages. Sensitivity, specificity, positive and negative predictive values, accuracy, likelihood ratios (LR) and 95% confidence intervals (CI) were calculated using the Twobytwo 1.0 analyzer program.

### Accession numbers

*Brucella*: bcsp31 (M20404), IS711 (AE017223)

MTC: IS6110 (BX842574), senX3-regX3 (BX842573).

## Results

**Figure 1:**
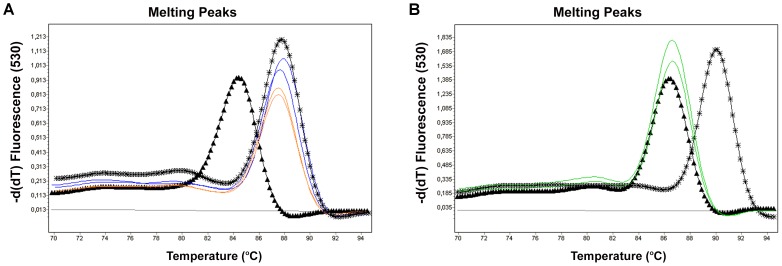
Specificity of PCR products based on the Tm. **Panel A**, M RT-PCR assay IS6110+IS711. Black triangles lines, positive control of *B. abortus*; Black stars, positive controls of *M. tuberculosis*; blue lines and orange lines, false positive results due to *M. intracellulare* and *M. simiae*, respectively. **Panel B**, M RT-PCR assay senX3-regX3+bcsp31. Black triangles lines, positive control of *B. abortus*; Black stars, positive controls of *M. tuberculosis*; green lines, false positive results related with *Ochrobactrum anthropi*.

The three M RT-PCR strategies based on amplification of the target sequences senX3-regX3+ bcsp31, senX3-regX3+ IS711 and IS6110+ IS711 correctly identified all the species and biovars of *Brucella* as well as all the species belonging to MTC. The target based on the IS711 sequence did not amplify any of the bacteria serologically or phylogenetically related with *Brucella* spp, and the amplicons of the genes of the bcsp31 protein gave a false positive result with *Ochrobactrum anthropi* and *intermedium*. Likewise, the amplicons of the intergenic region senX3-regX3 were negative in all NTM tested, but those of the IS6110 sequence amplified various NTM: *M. fortuitum, M scrofulaceum, M simiae* and *M. intracellulare* (Table 1). Figure 1 shows the similar melting temperatures (Tm) of two of these NTM (as an example) and the two strains of *Ochrobactrum* for bcsp31 compared with the control Tm for the two pathogens.

Of the 91 clinical samples included in the study, 35 were vertebral or paravertebral tissue from patients with vertebral osteomyelitis, 21 were CSF from patients with subacute or chronic meningitis or meningoencephalitis, 13 were tissue or abscess aspirates from patients with liver or splenic abscess, 8 were urine samples from patients with orchiepididymitis, 7 were from synovial fluid from patients with subacute or chronic arthritis, and the remaining 7 were from different locations: two samples of purulent fluid from patients with neck abscesses, two bone biopsies from patients with osteomyelitis of the femur and sternum, respectively, two kidney biopsies from patients with chronic pyelonephritis, and a sample of seminal fluid from a patient with chronic orchiepididymitis. Table 2 summarizes the sample type and the final diagnosis of the study patients.

**Table 2:** Sample type and diagnosis of the study patients.

Clinical Sample	Brucellosis	Tuberculosis	Control Group
	n (culture positive)	n (culture positive)	n
Vertebral or paravertebral tissue	11 (3)	12 (9)	12^a^
CSF	5 (1)	6 (4)	10^b^
Hepatic or splenic tissue	6 (1)	0	7^c^
Urine	4 (1)	2 (2)	2^d^
Synovial fluid	1 (1)	2 (2)	4^e^
Other samples	4 (1)	2 (1)	1^f^
**Total Samples**	**31**	**24**	**36**

a *S. aureus*, 4 cases, *E. coli* and *S. epidermidis* 2 cases, *S. agalactiae, Peptoestreptococcus*, *S. intermedius*, and *M. xenopi* one case respectively,

b Meningoencephalitis 4 cases (*T. whippelii*, V-Z, JC virus and toxoplasma), *criptococcu*s meningitis 2 cases and neurosyphilis, giant cell arteritis, neurosarcoidosis, meningeal carcinomatosis one case respectively,

c *E. coli* 4 cases, *P. aeruginosa*, *S. intermedius* and *B. fragilis* one case each respectively.

d *P. mirabilis* and seminoma one case respectively,

e *S. aureus* 3 cases and *N. meningitides* one case,

f Sternal osteomyelitis due to *Mycobacterium avium*.

Of the 30 patients with brucellosis, 25 (83.3%) were primary infections and 5 (16.6%) had had a previous episode of infection. Brucella melitensis was isolated in 17 (56.6%) of the 30 patients with brucellosis; 13 (43.3%) in blood culture, 8 (26.6%) in non-blood samples (three vertebral tissue and one each of the following: urine, CSF, hepatic tissue, synovial fluid and thyroid abscess) and in 4 (13.3%) in both blood and non-blood samples. In 12 of the other 13 patients (40%) the diagnosis of brucellosis was based on clinical and serological criteria. One 43-year-old woman, who habitually consumed non-homogenized dairy products and who had osteomyelitis with thoracic segment involvement and whose biopsy showed non-caseating granulomas, constantly had negative cultures and absence of serological response, and was diagnosed with brucellosis based on her epidemiologic exposure and clear response to treatment with doxycycline plus streptomycin.

Of the 24 patients with extrapulmonary tuberculosis, *M. tuberculosis* was isolated in 18 (75%) and the other six (25%) had necrotizing granulomas in their biopsies, with or without acid-fast bacilli. Only four (16.6%) of the 24 cases had smear-positive samples.

The three M RT-PCR strategies were positive in 49 (89.1%) of the 55 samples from patients with tuberculosis or brucellosis; 28 (90.3%) of the 31 focal complications of brucellosis and 21 (87.5%) of the 24 extrapulmonary tuberculosis. M RT-PCR was negative in the 36 samples from the control group patients. Thus, the overall sensitivity of the M RT-PCR was 89.1%, (95% CI, 80.9–97.3) and the specificity 100%. The overall diagnostic yield of the M RT-PCR is shown in Table 3.

**Table 3:** Diagnostic yield of M RT-PCR in clinical specimens from patients with focal complications of brucellosis or extrapulmonary tuberculosis.

	Sensitivity	Specificity	PPV	NPV	Accuracy	Positive LR	Negative LR
	%, (95% CI)						
All Samples	89.1, (80.9–97.3)	100	100	85.7, (75.1–96.3)	93.4, (88.3–96.5)	ND*	0.11,(0.05–0.23)
Focal Brucellosis	90.3, (79.9–100)	100	100	92.3, (83.9–100)	95.5, (90.6–100)	ND*	0.10, (0.03–0.28)
Extrapulmonary Tuberculosis	87.5, (74.3–100)	100	100	92.3, (83.9–100)	95.0, (89.5–100)	ND*	0.13, (0.04–0.36)

PPV, positive predictive value; NPV, negative predictive value; Positive LR, positive likelihood ratio; Negative LR, negative likelihood ratio; 95% CI = 95% confidence interval, ND*, not done for mathematical reasons (division by zero).

Of the six patients who had a false negative result with the M RT-PCR, two had received prolonged antimicrobial treatment before drawing the sample. The first of these was a 24-year-old woman with a kidney transplant and brucellar pyelonephritis in the transplanted organ, treated for two weeks before taking the renal biopsy with ciprofloxacin, meropenem and piperacillin-tazobactam. The second was a 33-year-old man with tuberculous vertebral osteomyelitis treated during the four months prior to taking the vertebral biopsy with rifampicin/isoniazid/pyrazinamide/ethambutol for the first two months and with rifampicin/isoniazid the second two months. In both cases the cultures were also negative. If these cases had been withdrawn from the analysis of efficacy, the sensitivity of the M RT-PCR for the overall sample would have risen to 92.5%. The other four false-negative results corresponded to two patients with brucellosis (one brucellar orchiepididymitis with positive blood cultures and a negative urine culture and the other vertebral osteomyelitis with negative blood and vertebral tissue cultures) and two patients with tuberculosis (one meningitis and one vertebral osteomyelitis, both with positive cultures and negative microscopic study). Table 4 shows the results of the M RT-PCR according to the type of microorganism, culture result and sample type. The M RT-PCR was positive in the four cases of extrapulmonary tuberculosis with smear-positive samples and in 17 (85%) of the 20 cases with smear-negative samples.

**Table 4:** Results of M RT-PCR according to clinical sample, microorganism, and culture result.

Clinical Sample	Brucellosis				Tuberculosis			
	Positive culture		Negative culture		Positive culture		Negative culture	
	M RT-PCR+,	MR T-PCR−	M RT-PCR+,	M RT-PCR−	M RT-PCR+,	M RT- PCR−	M RT-PCR+,	M RT-PCR−
Vertebral or paravertebral tissue	3	0	7	1	9	0	1	2
CSF	1	0	4	0	4	0	1	1
Hepatic or splenic tissue	1	0	5	0	0	0	0	0
Urine	1		0	2	1	2	0	0
Synovial fluid	1	0	0	0	1	0	1	0
Other samples	1	0	2	1	1	0	1	0
**Total Samples**	**8**	**0**	**20**	**3**	**17**	**0**	**4**	**3**

The mean Ct values of the senx3-regx3+ bcsp31, senx3-regx3+ IS711 e IS6110+ IS711 assays varied according to the type of sample, ranging from 31.03–34.85, 26.99–33.00 and 29.95–34.74 cycles respectively for the samples from patients with extrapulmonary tuberculosis to 24.68–30.57, 16.13–31.73 and 28.17–32.07 cycles for the samples from patients with focal complications of brucellosis.

**Figure 2:**
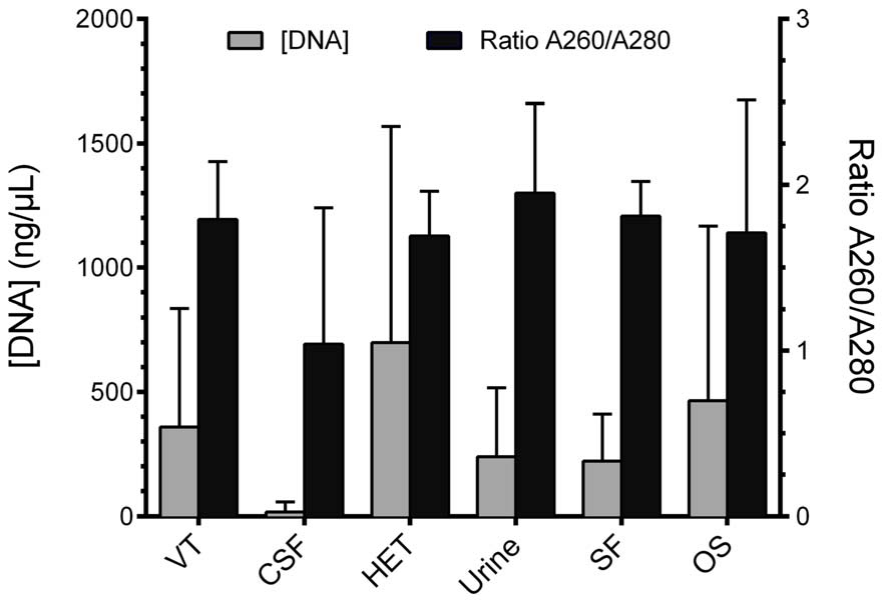
DNA concentrations and purity from the different clinical samples studied. Sample type: VT, vertebral or paravertebral tissue; CSF, cerebrospinal fluid; HET, hepatic or splenic tissue; SF, synovial fluid; OS, other samples.

The amount and purity of total DNA (microbial DNA and eukaryotic DNA) differed significantly depending on the type of clinical sample studied (Figure 2), though this did not affect the percentage of positive results with the test, independently of the M RT-PCR strategy used (Table 5).

**Table 5:** Tm (°C) and Ct (cycle) values with the three strategies of M RT-PCR studied by type of clinical sample.

Clinical samples	M RT-PCR											
	senX3-regX3+bcsp31				senX3-regX3+IS711				IS6610+IS711			
	*Brucella*		MTC		*Brucella*		MTC		*Brucella*		MTC	
	Tm	Ct	Tm	Ct	Tm	Ct	Tm	Ct	Tm	Ct	Tm	Ct
**Vertebral or paravertebral tissue**	86.64±0.30 (10)	30.57±6.32 (10)	90.07±0.43 (10)	32.39±5.67 (10)	84.58±0.54 (10)	30.07±5.32 (10)	90.00±0.36 (10)	31.66±6.29 (10)	84.56±0.19 (10)	31.03±1.95 (10)	87.64±0.66 (10)	31.42±6.78 (10)
**CSF**	86.88±0.32 (5)	28.31±5.34 (5)	90.03±0.61 (5)	32.68±3.33 (5)	84.69±0.27 (5)	28.58±7.98 (5)	90.21±0.36 (5)	26.99±4.36 (5)	84.28±0.20 (5)	32.07±2.67 (5)	87.62±0.24 (5)	32.14±1.04 (5)
**Hepatic or splenic tissue**	86.38±0.30 (6)	29.24±5.24 (6)	-	-	84.62±0.55 (6)	31.73±5.70 (6)	-	-	84.43±0.41 (6)	31.23±1.80 (6)	-	-
**Urine**	86.86±0.21 (3)	28.34±7.83 (3)	89.66±0.20 (2)	31.78±1.42 (2)	84.77±0.42 (3)	26.26±7.08 (3)	89.74±0.62 (2)	32.69±1.08 (2)	84.64±0.42 (3)	31.22±0.62 (3)	87.50±0.15 (2)	29.95±1.42 (2)
**Synovial fluid**	86.95 (1)	24.68 (1)	89.94±0.39 (2)	31.03±0.64 (2)	84.98 (1)	16.13 (1)	89.23±0.09 (2)	33.00±1.35 (2)	84.46 (1)	30.85 (1)	87.61±0.09 (2)	34.74±1.81 (2)
**Other samples**	86.51±0.31 (3)	26.85±2.95 (3)	90.50±0.15 (2)	34.85±1.32 (2)	84.54±0.59 (3)	26.11±1.79 (3)	90.32±0.36 (2)	29.62±4.85 (2)	84.23±0.34 (3)	28.17+3.20 (3)	87.72±0.09 (2)	32.21±5.30 (2)

Results are given as mean ± SD with the number of samples assayed in parentheses.

Finally, the ranges of the differences between the Tm of the PCR products of the clinical samples and the control strains were 0.02–0.63°C; a figure we consider irrelevant, and which is very expressive of the specificity of the technique (Table 6).

**Table 6:** Amplicons Tm of the three M RT-PCR assayed in different clinical samples and collection strains.

Type of sample assayed	M RT-PCR (Tm, °C)					
	senX3-regX3+bcsp31		senX3-regX3+IS711		IS6610+IS711	
	*Brucella* spp	MTC	*Brucella* spp	MTC	*Brucella* spp	MTC
Clinical samples (49)	86.64±0.33 (28)	90.05±0.48 (21)	84.64±0.46 (28)	89.98±0.44 (21)	84.45±0.30 (28)	87.62±0.46 (21)
Collection strains (32)	86.62±0.33 (22)	89.45±0.39 (10)	84.55±0.21 (22)	89.35±0.25 (10)	84.61±0.21 (22)	87.59±0.21 (10)
Difference of Tm (°C)	0.02	0.60	0.09	0.63	0.16	0.03

## Discussion

Since it was demonstrated that PCR can simultaneously amplify multiple loci of one or more different genes, multiplex PCR has become firmly established as a general technique [19]. As procedures become cheaper and simpler, molecular technology is being increasingly used in rapid microbiological diagnosis. Because of its high sensitivity, molecular diagnosis has now become a very useful tool for the diagnosis of many viral, bacterial and fungal infections.

Clinical microbiology is now directed more towards syndromic diagnosis, in which the most common causative agents of a particular clinical syndrome are all studied together at the same time in a single test. As M RT-PCR can do this ever more efficiently, it has experienced exponential development in recent years [14]–[16], [20], [21].

In many underdeveloped and developing countries, tuberculosis and brucellosis are still the most frequent causes of bacterial lymphocytic meningitis, granulomatous vertebral osteomyelitis, subacute arthritis and subacute orchiepididymitis. In these clinical scenarios, among others, M RT-PCR could be a useful tool for the rapid differential diagnosis between two pathogens whose isolation in culture is difficult and time consuming. Previous studies from our group have shown that of the different candidate genes, three combinations of amplicons of bcsp31 protein gene and the IS711 in the case of *Brucella* spp and the senX3-regX3 intergenic region and IS6110 for MTC permit a highly sensitive and reproducible co-amplification [18].

In this study we analyzed the diagnostic yield of the three possible combinations of the amplicons (senX3-regX3+ bcsp31, senX3-regX3+ IS711 and IS6110+ IS711) in a representative sample of patients with extrapulmonary tuberculosis and focal complications of brucellosis.

The three primer combinations correctly identified all the species and biovarieties of *Brucella* and MTC, and there was no non-specificity with the strategy based on the sequence amplification of senX3-regX3+ IS711. The target based on bcsp31 did, however, show a false positive result with *Ochrobactrum* spp. This cross-reaction, which has been described previously [22]–[23], is not surprising if we consider that *Ochrobactrum* spp. is the closest known relative of the Brucella genus. Concerning MTC, the target senX3-regX3 showed no non-specificity with the panel of NTM, though the strategy based on IS*6110* produced a cross-reaction with *M. fortuitum, M. scrofulaceum, M. intracellulare* and *M. simiae*. This lack of specificity has been previously described. Thus, a study analyzing the specificity of IS*6110*-based methods in nine laboratories from France demonstrated false-positive reactions with an average rate of 7%, most of them caused by NTM [24]. This explains why many authors request caution in designing and evaluating diagnostic PCR tests based on this element [25].

The overall sensitivity of our M RT-PCR method should be considered very good since it was 89.1%; 87.5% in extrapulmonary TB cases and 90.3% in cases of focal complications of brucellosis. These results are as good as or better than those with any of the monoplex PCR methods so far tried, sensitivities of which have ranged from 53–95% in clinical samples from patients with extrapulmonary tuberculosis [26]–[30] and from 92–94% for non-blood samples of focal complications of brucellosis [31].

The yield of molecular diagnostic techniques falls in patients with extrapulmonary tuberculosis with respiratory or nonrespiratory smear-negative specimens [26], [32].

In our study, only 4 (16.6%) of the 24 extrapulmonary tuberculosis cases were smear-positive, a percentage similar to that reported by other authors [26], [30]. This very small number of samples makes it difficult to draw conclusions about the sensitivity of our M RT-PCR assay in patients with extrapulmonary tuberculosis with smear-negative samples. Nevertheless, the results of this study (85% sensitivity in smear-negative samples) show the high sensitivity of the technique, even in paucibacillary specimens. This high sensitivity in smear-negative samples may be related with the fact that in our study most were aspirates from abscesses or tissue samples. Recently Moure *et al*, in a large study including 108 smear-negative extrapulmonary samples, found that the sensitivity of the Xpert was just 40.5% in sterile fluids versus 76.5% in abscess aspirates [33].

The diagnosis of brucellosis does not normally present problems in acute non-complicated forms. In these cases, all the serological tests commonly used have a high sensitivity. However, this is not the case in patients who have a more prolonged evolution, as occurs in most patients who have focal complications, particularly if they are patients who are professionally exposed or patients with recurrences of the disease. In both scenarios, serological studies have important limitations [34]. In addition, the sensitivity of the cultures, whether they are from peripheral blood or non-blood samples, does not usually surpass 50% in patients with focal forms of brucellosis. Other than our own studies, reports dealing with the usefulness of molecular techniques for the diagnosis of patients with focal complications of brucellosis are anecdotal, though they all show the superiority of these techniques as compared to cultures [35], [36].

In clinical practice the volume of a sample sent to the laboratory for the diagnosis of patients with extrapulmonary tuberculosis or focal brucellosis can vary greatly, depending on the site of the complication and the form of obtaining the sample. In fine-needle aspiration biopsies this volume can be really small. In our study the amount of DNA extracted and its purity can be considered good in all types of samples, except for CSF, as mentioned by others [37]. Concerning the amount and purity of DNA, previous studies by our group [18] have shown the inhibitory effect that high concentrations of DNA have on the technique. Given these previous results, in this study we used DNA amounts no greater than 250 ng per reaction, both in tissue samples and in abscesses. The small variable volumes of CSF available in clinical practice together with the peculiar characteristics of subacute lymphocytic bacterial meningitis ;mild or moderate pleocytosis, and paucibacillary samples meant that the volume of DNA for each assay varied, ranging between 2 and 8 µl for a final volume of 20 µl in the PCR reaction.

As is logical, the purity of the DNA differed widely depending on the type and location of the study sample, though this did not greatly affect the Ct or the Tm in comparison with what was seen in the collection strains of the two pathogens. From a qualitative point of view, neither the type of sample nor the amount or purity of the DNA influenced significantly the diagnostic yield of the M RT-PCR, independently of the strategy used, indicating the robustness of the three SYBR Green based M RT-PCR strategies.

Though the comparative study of the three pairs of amplicons used showed no differences in the samples used, the M RT-PCR strategy based on the amplification of senX3-regX3+ IS711 seems to be the most suitable, as it avoids false positive results derived not only from cross-reactions of IS6110 with NTM but also from amplification of *Ochrobactrum* spp., as this microorganism lacks IS711 [38].

The *Ochrobactrum* spp. comprises a group of very ubiquitous microorganisms. Although its ecology is not well known, it has been isolated from soil, water, multiple hospital material, and different clinical specimens and it may be part of the normal flora of the large intestine. *Ochrobactrum* spp. would seem to occupy a microbial niche similar to that of *Pseudomonas aeruginosa*, as most infections in humans have been in patients with catheters, other foreign bodies, or severely immunosuppressed persons [39]. Indeed, it is always important to exclude possible cross-reactions with potentially colonizing microorganisms.

In addition to its high sensitivity, other important aspects of single-tube M RT-PCR make it especially attractive to clinical laboratories for use in samples from patients in whom extrapulmonary TBC or focal complications of brucellosis are suspected. First, M RT-PCR provides results within 4 hours, which is much less than the time required for conventional methods to rescue a fastidious microorganism such as *M. tuberculosis* or *Brucella* spp; second, the technique almost completely obviates the need for direct handling of the pathogen, thus drastically reducing the risk of infection of laboratory personnel; and third, the sample can either be processed immediately or easily stored at −20°C until processing.

In conclusion, a SYBR Green single-tube M RT-PCR assay based on senX3-regX3+ IS711 coamplification allows a rapid and efficient identification of *M. tuberculosis complex* and *Brucella spp* in different clinical samples. Based upon our own experience with M RT-PCR and those of other authors, this new strategy is more specific than those previously reported, which, together with its high sensitivity, make it a very useful tool for the differential diagnosis between some forms of extrapulmonary tuberculosis and focal complications of brucellosis.

## Supporting Information

Checklist S1 STARD checklist for reporting of studies of diagnostic accuracy.
(DOC)
