# Supplementary material for: Comparative Clinical Study of Different Multiplex Real Time PCR Strategies for the Simultaneous Differential Diagnosis between Extrapulmonary Tuberculosis and Focal Complications of Brucellosis
Source: PLoS Negl Trop Dis. 2013 Dec 12;7(12):e2593. doi: 10.1371/journal.pntd.0002593 (PMC3861180; doi:10.1371/journal.pntd.0002593)
Supplement: Checklist S1 — STARD checklist for reporting of studies of diagnostic accuracy. (DOC) [file pntd.0002593.s001.doc]

# STARD checklist for reporting of studies of diagnostic accuracy

*(version January 2003)*

| **Section and Topic** | **Item**  **#** |  | **On page #** |
| --- | --- | --- | --- |
| TITLE/ABSTRACT/  KEYWORDS | 1 | Identify the article as a study of diagnostic accuracy (recommend MeSH heading 'sensitivity and specificity'). | **On page 2** |
| INTRODUCTION | 2 | State the research questions or study aims, such as estimating diagnostic accuracy or comparing accuracy between tests or across participant groups. | **On page 6** |
| METHODS |  |  |  |
| *Participants* | 3 | The study population: The inclusion and exclusion criteria, setting and locations where data were collected. | **On pages 6 and 7** |
|  | 4 | Participant recruitment: Was recruitment based on presenting symptoms, results from previous tests, or the fact that the participants had received the index tests or the reference standard? | **On page 7** |
|  | 5 | Participant sampling: Was the study population a consecutive series of participants defined by the selection criteria in item 3 and 4? If not, specify how participants were further selected. | The cases and the controls were all consecutive. |
|  | 6 | Data collection: Was data collection planned before the index test and reference standard were performed (prospective study) or after (retrospective study)? | The study design was retrospective |
| *Test methods* | 7 | The reference standard and its rationale. | **On pages 6 and 7** |
|  | 8 | Technical specifications of material and methods involved including how and when measurements were taken, and/or cite references for index tests and reference standard. | **On pages 8 and 9** |
|  | 9 | Definition of and rationale for the units, cut-offs and/or categories of the results of the index tests and the reference standard. | **On page 8**  **and (reference 18)** |
|  | 10 | The number, training and expertise of the persons executing and reading the index tests and the reference standard. | The index test was done by one of the authors and the reference tests by clinical microbiologists from the participating hospital. |
|  | 11 | Whether or not the readers of the index tests and reference standard were blind (masked) to the results of the other test and describe any other clinical information available to the readers. | **On page 9** |
| *Statistical methods* | 12 | Methods for calculating or comparing measures of diagnostic accuracy, and the statistical methods used to quantify uncertainty (e.g. 95% confidence intervals). | **On pages 10 and 27 (Table3)** |
|  | 13 | Methods for calculating test reproducibility, if done. | **On pages 8 (reference 18)** |
| RESULTS |  |  |  |
| *Participants* | 14 | When study was performed, including beginning and end dates of recruitment. | The scarce incidence of the target diseases makes fixing start and end dates of recruitment impossible.. |
|  | 15 | Clinical and demographic characteristics of the study population (at least information on age, gender, spectrum of presenting symptoms). | **On pages 6, 10, 11 and 12** can be found the suitable clinical information |
|  | 16 | The number of participants satisfying the criteria for inclusion who did or did not undergo the index tests and/or the reference standard; describe why participants failed to undergo either test (a flow diagram is strongly recommended). | **On page 11** |
| *Test results* | 17 | Time-interval between the index tests and the reference standard, and any treatment administered in between. | **On pages 8 and 11** |
|  | 18 | Distribution of severity of disease (define criteria) in those with the target condition; other diagnoses in participants without the target condition. | **On pages 10, 11 and 26 (Table 2)** |
|  | 19 | A cross tabulation of the results of the index tests (including indeterminate and missing results) by the results of the reference standard; for continuous results, the distribution of the test results by the results of the reference standard. | **On pages 28 (Table 4)** |
|  | 20 | Any adverse events from performing the index tests or the reference standard. | Due to the nature of the study the samples were obtained from different body sites, though there were no complications regarding obtaining these samples. |
| *Estimates* | 21 | Estimates of diagnostic accuracy and measures of statistical uncertainty (e.g. 95% confidence intervals). | **On page 27 (Table 3)** |
|  | 22 | How indeterminate results, missing data and outliers of the index tests were handled. | There were no indeterminate results or missing data in the study |
|  | 23 | Estimates of variability of diagnostic accuracy between subgroups of participants, readers or centers, if done. | **On pages 26, 27 and 28 (Tables 2, 3 and 4)**  The whole study was undertaken at one single centre |
|  | 24 | Estimates of test reproducibility, if done. | **On page 8 (reference 18)** |
| DISCUSSION | 25 | Discuss the clinical applicability of the study findings. | **On pages 14, 15, 16 and 17** |
